# T-cell responses in colorectal peritoneal metastases are recapitulated in a humanized immune system mouse model

**DOI:** 10.3389/fimmu.2024.1415457

**Published:** 2024-07-09

**Authors:** Job Saris, Sanne Bootsma, Jan Verhoeff, Jurriaan B. Tuynman, Manon E. Wildenberg, Esther Siteur-van Rijnstra, Kristiaan J. Lenos, Juan J. Garcia Vallejo, Louis Vermeulen, Joep Grootjans

**Affiliations:** ^1^ Department of Gastroenterology and Hepatology, Amsterdam UMC location University of Amsterdam, Amsterdam, Netherlands; ^2^ Amsterdam Gastroenterology Endocrinology Metabolism, Amsterdam, Netherlands; ^3^ Cancer Center Amsterdam, Amsterdam, Netherlands; ^4^ Tytgat Institute for Liver and Intestinal Research, Amsterdam UMC location University of Amsterdam, Amsterdam, Netherlands; ^5^ Oncode Institute, Amsterdam, Netherlands; ^6^ Center for Experimental and Molecular Medicine, Laboratory for Experimental Oncology and Radiobiology, Amsterdam UMC location University of Amsterdam, Amsterdam, Netherlands; ^7^ Molecular Cell Biology & Immunology, Amsterdam UMC location Vrije Universiteit, Amsterdam, Netherlands; ^8^ Amsterdam Infection & Immunity Institute, Amsterdam, Netherlands; ^9^ Department of Surgery, Amsterdam UMC location Vrije Universiteit Amsterdam, Amsterdam, Netherlands; ^10^ HIS Mouse Facility, Amsterdam UMC Location University of Amsterdam, Amsterdam, Netherlands

**Keywords:** colorectal cancer, peritoneal metastasis, peritoneal immune system, humanized immune system, T-cell biology, CyTOF

## Abstract

**Background:**

The occurrence of peritoneal metastasis (PM) in patients with colorectal cancer (CRC) has a dismal prognosis. There is often limited response to systemic- and immunotherapy, even in microsatellite unstable (MSI) CRC. To overcome therapy resistance, it is critical to understand local immune environment in the peritoneal cavity, and to develop models to study anti-tumor immune responses. Here, we defined the peritoneal immune system (PerIS) in PM-CRC patients and evaluate the pre-clinical potential of a humanized immune system (HIS) mouse model for PM-CRC.

**Methods:**

We studied the human PerIS in PM-CRC patients (n=20; MSS 19/20; 95%) and in healthy controls (n=3). HIS mice (NODscid gamma background; n=18) were generated, followed by intraperitoneal injection of either saline (HIS control; n=3) or human MSS/MSI CRC cell lines HUTU80, MDST8 and HCT116 (HIS-PM, n=15). Immune cells in peritoneal fluid and peritoneal tumors were analyzed using cytometry by time of flight (CyTOF).

**Results:**

The human and HIS mouse homeostatic PerIS was equally populated by NK cells and CD4+- and CD8+ T cells, however differences were observed in macrophage and B cell abundance. In HIS mice, successful peritoneal engraftment of both MSI and MSS tumors was observed (15/15; 100%). Both in human PM-CRC and in the HIS mouse PM-CRC model, we observed that MSS PM-CRC triggered a CD4+ Treg response in the PerIS, while MSI PM-CRC drives CD8+ TEMs responses.

**Conclusion:**

In conclusion, T cell responses in PM-CRC in HIS mice mirror those in human PM-CRC, making this model suitable to study antitumor T cell responses in PM-CRC.

## 1 Introduction

Peritoneal metastases (PMs) in colorectal cancer (CRC) pose a significant clinical challenge due to extensive morbidity and poor prognosis (1). The vast majority of PM-CRC classify as consensus molecular subtype 4 (CMS4) (2, 3), a well-established disease subtype that is associated with increased cell motility and poor prognosis (4). Most cancers of this subtype are microsatellite stable (MSS), and harbor a proficient DNA mismatch repair (MMR) system, typically resulting in low antigen presentation and limited response to immune checkpoint blockade (ICB) therapy. In contrast, microsatellite unstable (MSI) cancers, caused by silencing or mutational inactivation of MMR genes, have shown remarkable responses to ICB therapy (5–7). However, even within the subgroup of MSI CRC, the presence of PM is a negative predictor for response to ICB (8). As the PerIS harbors a diverse population of immune cells (9), we hypothesize that the peritoneal immune system (PerIS) contributes to the tumor immune microenvironment (TIME) in PM-CRC. To effectively translate pre-clinical studies to patients with peritoneal metastatic disease, it is important to develop clinically-relevant *in vivo models* that mimic the complexity of both human cancer cells and the human PerIS. In such models, human anti-tumor T cell responses to human cancer cells can be studied in detail which may help in improving current therapies for PM-CRC.

Genetically engineered or syngeneic mouse cancer models are commonly used to study the TIME and immunotherapy *in vivo*. However, they often fall short in translating to the human setting (10) and genetically engineered mouse models that develop spontaneous peritoneal metastasis are lacking to the best of our knowledge. To overcome this limitation, humanized immune system (HIS) mice have been developed, wherein human hematopoietic stem cells are injected into immunodeficient mice, which subsequently mature into human immune cells. While HIS mice have been used to study immune modulatory therapies in CRC (11, 12), immune profiling in the peritoneal metastasized setting is lacking. In particular, the T cell compartment within the PerIS in humans is understudied and the composition of the PerIS in HIS mice is unknown. In this study, we used high throughput mass cytometry to comprehensively characterize the PerIS in humans, as well as the PerIS and TIME in a PM-CRC HIS mouse model.

## 2 Material and methods

### 2.1 Cell culture

Cell lines HUTU80 and MDST8 were cultured in Dulbecco’s modified Eagle’s medium/F-12 medium with L-glutamine, 15 mM HEPES (Thermo-Fisher Scientific) supplemented with 8% fetal bovine serum (Life Technologies), penicillin and streptomycin. HCT116 was cultured in Roswell Park Memorial Institute (RPMI) 1640 with L-glutamine, 25 mM HEPES (Thermo-Fisher Scientific) supplemented with 8% fetal bovine serum (Life Technologies), penicillin and streptomycin, 1% D-glucose solution plus (Sigma-Aldrich) and 100 μM sodium pyruvate (Thermo-Fisher Scientific). All cell lines were obtained from the Sanger Institute (Cambridge, UK), authenticated by STR Genotyping and regularly tested for mycoplasma infection.

### 2.2 Animal experiments

All *in vivo* experiments were approved by the animal experimentation committee at the Amsterdam UMC (location Academic Medical Center (AMC) in Amsterdam under the nationally registered license numbers AVD118002016493 and AVD11800202013801) and performed according to national guidelines. NOD.Cg-Prkdc^scid^ Il2rg^tm1Wjl^/Szj (NSG) mice were bred in-house.

### 2.3 Establishment of HIS-NSG mice

HIS-NSG mice were generated by injecting human embryonic hematopoietic stem cells into five-day-old mice. Newborn mice were sub-lethally irradiated once (1 Gy) using a ^137^Cs source and human CD34^+^CD38^−^lineage^−^ cells (5 × 10^4^ cells) were intrahepatically injected. Eight weeks later, peripheral blood was collected from the submandibular vein to determine the reconstitution of a HIS. Successful humanization was assessed by determining the human immune cell engraftment score (>20%). Mice were housed in individually ventilated cages with sterile bedding, food, and acidified water ad libitum.

### 2.4 Peritoneal tumor growth

To generate intraperitoneal tumors, 5 x 10^4^ colon cancer cells in medium were mixed at a 1:1 ratio with Matrigel (Corning) and injected intraperitoneally in HIS-NSG mice. Four to five weeks after injection, mice were euthanized and peritoneal lavage with 5 ml ice cold FACS buffer (PBS, 2%FCS, 0,1mM EDTA) was performed. Tumors were harvested and scored according to the modified Peritoneal Carcinomatosis Index (PCI) score as described previously (13).

### 2.5 Patient cohort

Both HC and PM-CRC patients were enrolled in the study according to Dutch research guidelines of the Federation of Dutch Medical Scientific Societies (FMDSS), as described in “Human Tissue and Medical Research: Code of Conduct for Responsible use”. Patients were informed and provided informed consent prior to sampling. Patients did not receive any form of compensation. All patients underwent laparoscopic or laparotomic abdominal surgery for diagnostic or therapeutic purpose. In total, 23 patients (3 achalasia and 20 PM-CRC) enrolled in this study at the Amsterdam University Medical Centers, locations AMC and VUmc between 2019 and 2022. To study the peritoneal immune system in homeostasis, patients that underwent surgery for achalasia (n=3), a rare disorder of the esophagus, in which no immunological alterations of the peritoneal cavity are to be expected, were included. In these patients, there is access to the peritoneal cavity to perform peritoneal flushes, yet these patients do not have peritoneal involvement of disease. For PM-CRC, the inclusion criteria were as follows: Histologically proven colorectal carcinoma with histological proven peritoneal metastasis of which 95% was MSS (19/20). Patients were aged 18 years and older and did not have other intra-abdominal diseases or complaints (i.e. endometriosis, pancreatitis, appendicitis, cholecystitis, cholangitis, perforation of the GI tract, bleeding of the GI tract or corpus alienum). Patients who had underwent abdominal surgery shorter than 6 months ago were excluded from the study.

### 2.6 Sample collection

Samples were obtained perioperatively during abdominal surgery. To ensure stringent patient inclusion and tissue sampling, researchers were always present during surgery. Access to the abdominal cavity was performed with minimal blood contamination after which the peritoneal cavity was flushed with 1 liter of 0,9% NaCl saline solution (body temperature). The flush was infused with standard irrigation/suction device and aimed towards the diaphragm right (1/3), diaphragm left (1/3) and omentum and paracolic gutters (1/3). Two minutes after infusion the peritoneal flush was removed by suction and saved in clean plastic canisters. The peritoneal fluid was then quickly transferred to a glass canister and kept on ice until further processing. Peritoneal tumor samples were taken perioperatively at discretion of the operating surgeon and transferred to transferring medium (RPMI 1640 + 10% FCS) for processing into single cell suspensions.

### 2.7 Tissue dissociation and preparation for CyTOF

To isolate single cells for CyTOF, tumors were transferred to a 6-well plate and 3 ml warm digestion medium (RPMI 1640, 1.5 mg/ml Collagenase, 20 µg/ml DNAse I) per well was added. Tumors were cut into small pieces using a scalpel blade and placed at 37°C for 30 min (mouse) or 45 min (human) with a magnetic stirrer on a magnetic plate. To stop the digestion process, 3 ml cold wash medium (RPMI 1640 + 8%FCS) was added, the suspension was forced through a 70 µm cell strainer (Greiner) and spun down at 1500 rpm for 7 min. The pellet was resuspended in 2 ml red blood cell lysis buffer (eBioscience) and incubated for 5 min at RT. Eight ml cold wash medium was added and the suspension was spun down at 1500 rpm for 7 min and washed twice and resuspended in FACS buffer (PBS + 0.1% BSA).

Spleens were pushed through a 100 µm cell strainer (Greiner) with a syringe plunger and spun down at 1500 rpm for 7 min. The pellet was resuspended in 5 ml red blood cell lysis buffer (eBioscience) and incubated for 5 min at RT. Ten ml FACS buffer (PBS + 0.1% BSA) was added and the suspension was forced through 70 µm cell strainer (Greiner). After spinning at 1500 rpm for 7 min, the sample was resuspended in FACS buffer (PBS + 0.1% BSA). Tumor and spleen cells were counted and brought to a concentration of 25 x 10^6^/ml for subsequent sorting.

### 2.8 Flow cytometry

Cells were stained with anti-human CD45 AF700 (Biolegend) for 30 min at 4°C and washed twice. DAPI^-^CD45^+^ cells were sorted using the SH800 Cell Sorter (Sony). Only samples with sufficient yield were eligible for further analysis.

### 2.9 CyTOF

After successful isolation of single cells and counting of the cells, a maximum of 4 million cells were washed with 5 mL of Maxpar PBS and spun down at 1500 rpm for 7 min. Live/dead staining was performed with Cell-ID™ cisplatin (5uM) (Standard BioTools) in Maxpar PBS (Standard BioTools) and incubated for 5 min on RT. The cells were washed once with 1mL of Maxpar Cell Stain buffer (CSB) (Standard BioTools) and spun down at 1500 rpm for 5 min. For fixation and subsequent barcoding, cells were fixed with 1,6% paraformaldehyde (PFA) in Maxpar PBS and incubated for 10 min at RT. The cells were washed twice with Barcode Perm Buffer (Standard BioTools) and spun down at 1850rpm for 5 min. Thereafter, cells were resuspended in 100ul Barcode Perm Buffer and 10µl of the allocated barcode (Cell-ID™ 20-Plex Pd Barcoding Kit) was added and samples were incubated for 30min at RT. Finally, the samples were washed twice with CSB and frozen for long term storage in FCS 10%DMSO using Mr Frosty™ (Thermo-Fisher Scientific) at -80°C.

At the day prior to acquisition on the Helios™ CyTOF system (Standard BioTools), cells were thawed on ice and all barcoded samples were pooled into one 50mL tube with addition of 1:1 CSB (mL). After spinning down the pooled samples 800g for 7min, the supernatant was removed and 10mL prewarmed 37°C CSB with DNAse (150U/mL) was added and incubated for 10min at RT. After washing and counting the cells, Human Trustain FcX™ Fc blocking reagent (Biolegend) was added and cells were incubated for 10 min at RT and spun down 800g 7 min. Then surface staining antibody cocktail in CSB was subsequently added to the cells and incubated for 30 min at RT. Afterwards, the cells were spun down and washed twice in Perm-S buffer (Standard BioTools), after which the intracellular antibody cocktail (in Perm-S buffer) was added and incubated for 30 min at RT. The sample were again washed twice with CSB and fixed with 1,6% fresh PFA for 10 min at 4°C. Samples are spun down 800g for 5min and 125nM Cell-ID Iridium (Standard Biotools) was added in Fix and Perm buffer (Standard BioTools) and incubated overnight at 4°C. The complete CyTOF antibody panel is listed in **Supplementary Table 2**.

When cell yield of an experimental batch was greater than daily throughput the sample was split into two parts and acquired on subsequent days. At the day of acquisition, the aliquot of cells is washed twice with RPMI 1640 (Thermo-Fisher Scientific) 30% FCS and spun down 800g for 5 min. The cells are washed with CSB and counted. The cells then are divided into smaller aliquots of 1-2 million cells and prior to acquisition the cells are washed again with CSB and one final wash with Cell Acquisition Solution (CAS) (Standard BioTools) before proceeding towards the Helios™ CyTOF system. Aliquots were resuspended in CAS to a concentration of 0.8-1 x 10^6^ cells/ml with 10% (v/v) of 4-element EQ beads (Standard Biotools) for signal normalization over time.

Resulting fcs files were normalized over time and debarcoded using CyTOF software version 6.7 per manufacturer’s instructions. Signal over time was monitored for consistent flow and disrupted signal due to clogs was gated out. Events per sample were gated in OMIQ (Dotmatics) to singlets using Gaussian parameters and DNA content (iridium signal) (14). Live platinum negative singlets were used for subsequent analysis in R version 3.6 (R Core team). Each experimental batch contained a PerIS technical replicate sample from previous batches. Technical variation between batches was minimized using CytoNorm based on this technical replicate (15). After normalization CD45^+^ cells were phenotyped in OMIQ through gating after optSNE dimensionality reduction based on known expression patterns. Cut-off to distinguish cells positive or negative for activation/exhaustion markers (PD-1, CD69) were determined based on internal controls (e.g., no positive expression expected of PD-1 on monocytes).

### 2.10 Immunohistochemistry

Directly after isolation, tumors were fixed in 4% paraformaldehyde overnight prior to paraffin embedding. Tissue sections (5 μm) were deparaffinized and antigen retrieval was performed using 10 mM sodium citrate and boiling for 20 min. Endogenous peroxidase activity was blocked with 3% hydrogen peroxide in PBS. A specific staining was blocked using UltraVision Protein Blk (Thermo Scientific, Waltham, MA) 10 min on RT. Primary antibodies CD4 (Abcam, 1:50), CD8 (DAKO, 1:50), CD20 (DAKO, 1:500), CD68 (DAKO, 1:10,000) and PD1 (Cell Marque Corporation, 1:100) were diluted in antibody diluent (Agilent: CD4, PD1; Ventana: CD8, CD20, CD68) and incubated in a humidified chamber according to manufacturer’s protocol. For amplification of the staining, Brightvision+ post antibody block (Immunologic) was used for 20 min prior to the addition of the secondary antibody, poly-HRP-anti Ms/Rb IgG (Immunologic) for 30 min at RT. Visualization of stainings was performed with Bright DAB solution (Immunologic) according to manufacturer’s protocol, counterstained with undiluted Mayer Haematoxylin (Klinipath) and mounted tissue sections with non-aqueous medium. Positive cells were automatically counted using QuPath software version 0.3.2.

### 2.11 Statistical analysis

Statistical significance was determined as indicated in the figure legends. Differences were considered significant at *P* < 0.05. Data were analyzed using GraphPad Prism v9 (GraphPad Software). For between-group comparisons, a non-parametric Mann-Whitney (MW) test was used if groups were not normally distributed.

Every immune subset was calculated as a percentage of total CD45^+^ cells, or in the case of T cell subsets, as percentage of parent population or total T cell population.

## 3 Results

### 3.1 Increase of CD4 regulatory T cells in the peritoneal immune system of PM-CRC patients

**Figure 1:**
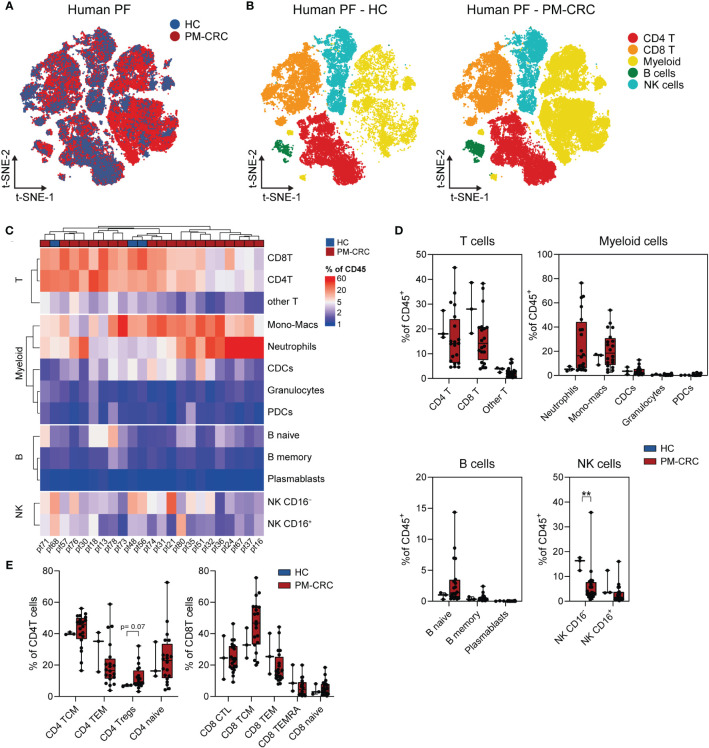
The human PerIS is characterized by abundant CD4^+^- and CD8^+^ T cells, NK cells and myeloids. **(A)** t-SNE overlay of human PF color coded per condition: HC (blue) and PM-CRC (red) showing the overlap between both groups. **(B)** t-SNE of human PF color coded per immune subset (n=5) and split by condition: HC (left) and PM-CRC (right) identifying major immune subsets in both groups. **(C)** Complex unsupervised heatmap showing proportion of immune subsets in the PerIS relative to CD45^+^ immune cells, grouped per main lineage, of human HCs (n=3) and PM-CRC patients (n=20). Samples did not cluster based on HC/PM-CRC status. **(D)** Boxplot of human HC PF (n=3) vs PM-CRC (n=20) showing proportion of immune subsets in the PerIS relative to CD45^+^ immune cells, grouped per main lineage showing the large abundance of CD4- and CD8 T cells in both conditions. **(E)** Boxplot comparison of T cells in human HC PF (n=3) compared to human PM-CRC PF (n=20) showing proportion of CD4- or CD8 immune subsets in the PerIS relative to CD4- or CD8 T cells, respectively. Showing increase of CD4^+^ Tregs in PM-CRC patients. Statistics: Mann-Whitney U test. HC, healthy controls; PM-CRC, peritoneal metastasized colorectal cancer; PF, peritoneal fluid; CyTOF, cytometry by time of flight; t-SNE, t-Distributed Stochastic Neighbor Embedding; CD4 T, CD4^+^ T cells; CD8 T, CD8^+^ T cells; Other T, double negative T cells and/or double positive T cells; mono-macs, monocyte/macrophages; CDCs, conventional dendritic cells; PDCs, plasmacytoid dendritic cells; NK, natural killer cell; B, B cells; TCM, T central memory; TEM, T effector memory; Tregs, regulatory T cells; CTL, cytotoxic T cells; TEMRA, Terminally differentiated effector memory. Whiskers show minimum and maximum data values. ** = p ≤ 0.01.

To establish a thorough characterization of the PerIS in humans, we used CyTOF technology enabling a 36-surface protein panel suited for deep immune phenotyping. The PF of both healthy controls (n=3; 323.886 cells) as well as PM-CRC (n=20; 5.407.642 cells; 19/20 (95%) MSS) patients was analyzed (**Supplementary Figure 1A**; **Supplementary Table 1**). Distributed stochastic neighbor embedding (t-SNE) of PF of both HC and PM-CRC patients combined reveal a number of major immune subsets including CD4^+^ T cells, CD8^+^ T cells, myeloid cells, B cells and natural killer (NK) cells (**Figures 1A, B**). Interestingly, unsupervised clustering of HC and PM-CRC patients shows that the majority of the PF in HC and PM-CRC patients contains CD4T- and CD8T cells, monocyte/macrophages (mono-macs), and NK CD16^–^ cells. Conversely, numbers of plasmacytoid dendritic cells (pDCs), B cells and granulocytes are low within the human PerIS (**Figure 1C**). Proportionally, most of the immune subsets within the PerIS do not change considerably upon presence of peritoneal metastasis. Indeed, when comparing HC vs PM-CRC, CD4^+^- (mean 20,7% vs 16,2%) and CD8^+^ T cells (mean 28,3% vs 16,1%), remain the largest population of cells, respectively. We observed a trend towards an increase in neutrophils (mean 5,4% vs 26,4%), which indicates an inflammatory response to PM-CRC, similar to what has previously been described (16). Similar proportions are found in other myeloid subpopulations when comparing HC vs PM-CRC: mono-macs (mean 14,3% vs 21,3%), conventional dendritic cells (CDCs; mean 3,7% vs 3,7%), granulocytes (mean 0,6% vs 0,5%) and plasmacytoid DCs (pDCs; mean 0,2% vs 0,5%), respectively. There were very few B naive, B memory and plasmablasts present in the normal human PerIS (each <1%), with only a slight increase in B naive cells in PM-CRC (mean 3%). Finally, we observed a significant decrease in CD16^–^ (mean 15,4% vs 6,0%), but not in CD16^+^ (mean 6,4% vs 3,1%) NK cells comparing HC vs PM-CRC, respectively (**Figure 1D**). As T cells are a dominant cell population within the PerIS in humans and plays a central role in current ICB strategies, this immune subset was subject to further research. Further classification of T cells subsets using canonical marker expression (**Supplementary Figure 1B**) shows a trend towards an increase of CD4^+^ regulatory T cells (CD4^+^ Tregs) (7,0% vs 12,1%; p = 0.07), comparing HC to PM-CRC respectively (**Figure 1E**).

This data provides an overview of the main immune subsets in the PerIS in healthy human subjects as well as those suffering peritoneal metastasized cancer. In particular, CD4^+^ Tregs within the PerIS are increased in individuals with peritoneal metastasized colorectal cancer.

### 3.2 Cell line dependent human-like immune response in the peritoneal cavity of HIS mice

**Figure 2:**
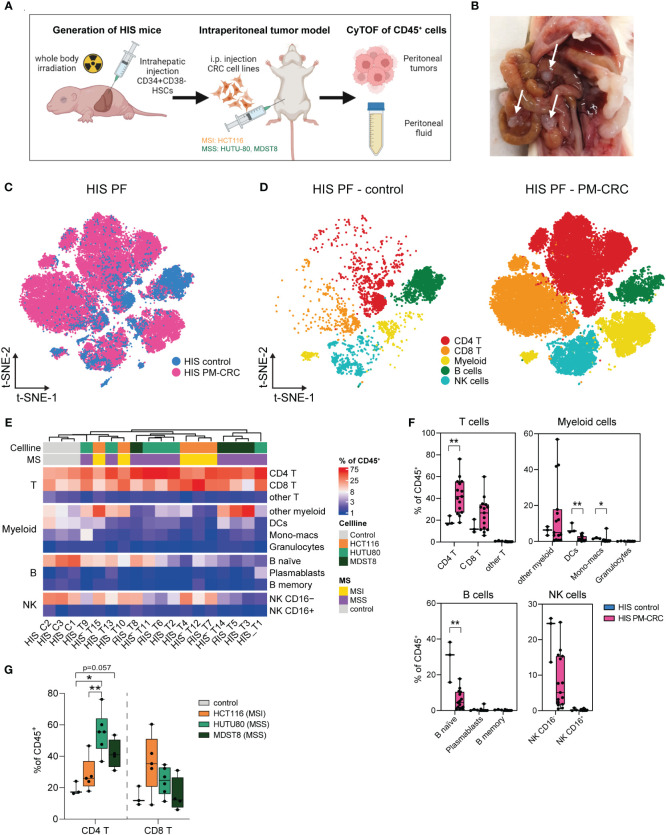
Tumor dependent human-like immune response in the peritoneal cavity of HIS mice. **(A)** Schematic workflow of HIS intraperitoneal sample collection. HIS mice were injected intraperitoneally with human CRC cell lines (50.000 cells/injection) HCT116 (CMS1, MSI), HUTU80 and MDST8 (both CMS4, MSS). Peritoneal fluid and tumors were collected, digested, sorted for CD45^+^ cells and analyzed using CyTOF. **(B)** Representative picture of peritoneal tumors of MDST8 cells on the mesentery of a HIS mouse. Arrows depict tumors. **(C)** t-SNE overlay of HIS PF color coded per condition: HIS control (blue) and HIS PM-CRC (pink) identifying presence of major immune subsets in both groups. **(D)** t-SNE of HIS PF color coded per immune subset (n=5) and split by condition: HIS control without tumor injection (left) and HIS PM-CRC with tumor injection (right) identifying five immune subsets in both groups. **(E)** Complex unsupervised heatmap showing proportion of immune subsets in the PerIS relative to CD45^+^ immune cells, grouped per main lineage, of HIS control mice (n=3) and HIS tumor mice (n=15). Colors indicate injected cell line and MS status. **(F)** Boxplot comparison of HIS control mice (n=3) and HIS PM-CRC mice (n=15) showing proportion of immune subsets in the PerIS relative to CD45^+^ immune cells, grouped per main lineage. An increase in CD4 T cells and a decrease in B naive cells was observed in PM-CRC compared to control. Statistics: Mann-Whitney U test. **(G)** Boxplot comparison of the proportion of CD4 T and CD8 T cells in the PerIS relative to CD45^+^ immune cells in HIS control mice (n=3), and mice injected with HCT116 (n=5), HUTU80 (n=6) and MDST8 (n=4). Increase of CD4 T cells in HUTU80 and MDST8. Statistics: Mann-Whitney U test. HIS, humanized immune system; HSCs, hematopoietic stem cells; t-SNE, t-Distributed Stochastic Neighbor Embedding; PF, peritoneal fluid; PM-CRC, peritoneal metastasized colorectal cancer; MS, microsatellite; MSI, microsatellite instable; MSS, microsatellite stable; CD4 T, CD4^+^ T cells; CD8 T, CD8^+^ T cells; Other T, double negative T cells and/or double positive T cells; DCs, dendritic cells; NK, natural killer cell. Whiskers show minimum and maximum data values. (* = p ≤ 0.05; ** = p ≤ 0.01).

To establish a humanized immunocompetent PM-CRC model, we intraperitoneally injected either CMS1 (HCT116, MSI) or CMS4 (MDST8 or HUTU80, MSS) cell lines into HIS mice and sacrificed them 4-5 weeks after tumor cell injection to assess peritoneal tumor growth and immune cell composition (**Figures 2A, B**). Successful engraftment and subsequent tumor formation was observed in all regions of the peritoneal cavity, with the omentum and the mesentery being particularly affected (**Supplementary Figure 2A**). Tumors from cell line HUTU80, and to a lesser extent HCT-116, caused ascites formation in mice (**Supplementary Figure 2A**). To better understand the impact of peritoneal tumors on the immune cell subsets in the PF, we immune profiled the PF of HIS mice without (HIS control; n=3; 7.341 cells) and with (HIS tumor; n=15; 521.803 cells) peritoneal tumors (**Figures 2C, D**). Deep immunophenotyping analyses were performed on viable DAPI^–^CD45^+^ human immune single cells which were isolated from the peritoneal fluid (**Supplementary Figures 2B, C**). Neither cell line, nor HIS immune donor mix was responsible for unique immune cluster formation (**Supplementary Figures 2D, E**). Interestingly, the presence of peritoneal tumors had a noticeable effect on the immune composition of the PF. We observed a proportional decrease in B naïve cells (mean 28,4% vs 5,8%) and NK CD16^–^ cells (mean 21,4% vs 8,1%), along with a significant increase in CD4^+^- (mean 19,2% vs 42,6%) and CD8^+^ T cells (mean 13,9% vs 25,5%), comparing HIS control vs HIS PM-CRC, respectively (**Figures 2E, F**). Within the myeloid population, a unique and HIS-exclusive immune subset was identified, which we referred to as ‘other myeloid’ (**Figures 2E, F**; **Supplementary Figures 2F, G**). This subset displayed an atypical combination of canonical marker expression (HLA-DR^+^CD11c^+^CD123^+^CD163^–^CD206^–^), but resembled best human macrophages based on unsupervised hierarchical clustering (**Supplementary Figure 2H**). Given the vast increase of T cells, we further explored this immune population. There were no differences in CD4^+^- and CD8^+^ T cell proportions in both human and HIS controls (**Supplementary Figures 2I**). Interestingly, the PerIS showed markedly different immune responses between the different cell lines that were injected. In the presence of MSS cell lines HUTU80 and MDST8 the PerIS contained more CD4^+^ T cells (mean 55.3% and 41.6, respectively) compared to HIS control mice (mean 19,2%) and HIS mice injected with MSI cell line HCT116 (mean 28,3%) (**Figure 2G**). Furthermore, CD8^+^ T cells are mainly increased in HCT116 (mean 35,5%) as compared to control HIS (mean 13,9%) (**Figure 2G**). Altogether, both MSI and MSS cell lines successfully engraft in the peritoneum of HIS mice. Peritoneal immune responses in the PerIS of HIS mice are primarily T cell driven and different between cell lines.

### 3.3 Tumor MSS/MSI status define the T cell infiltrate in PM-CRC of HIS mice

**Figure 3:**
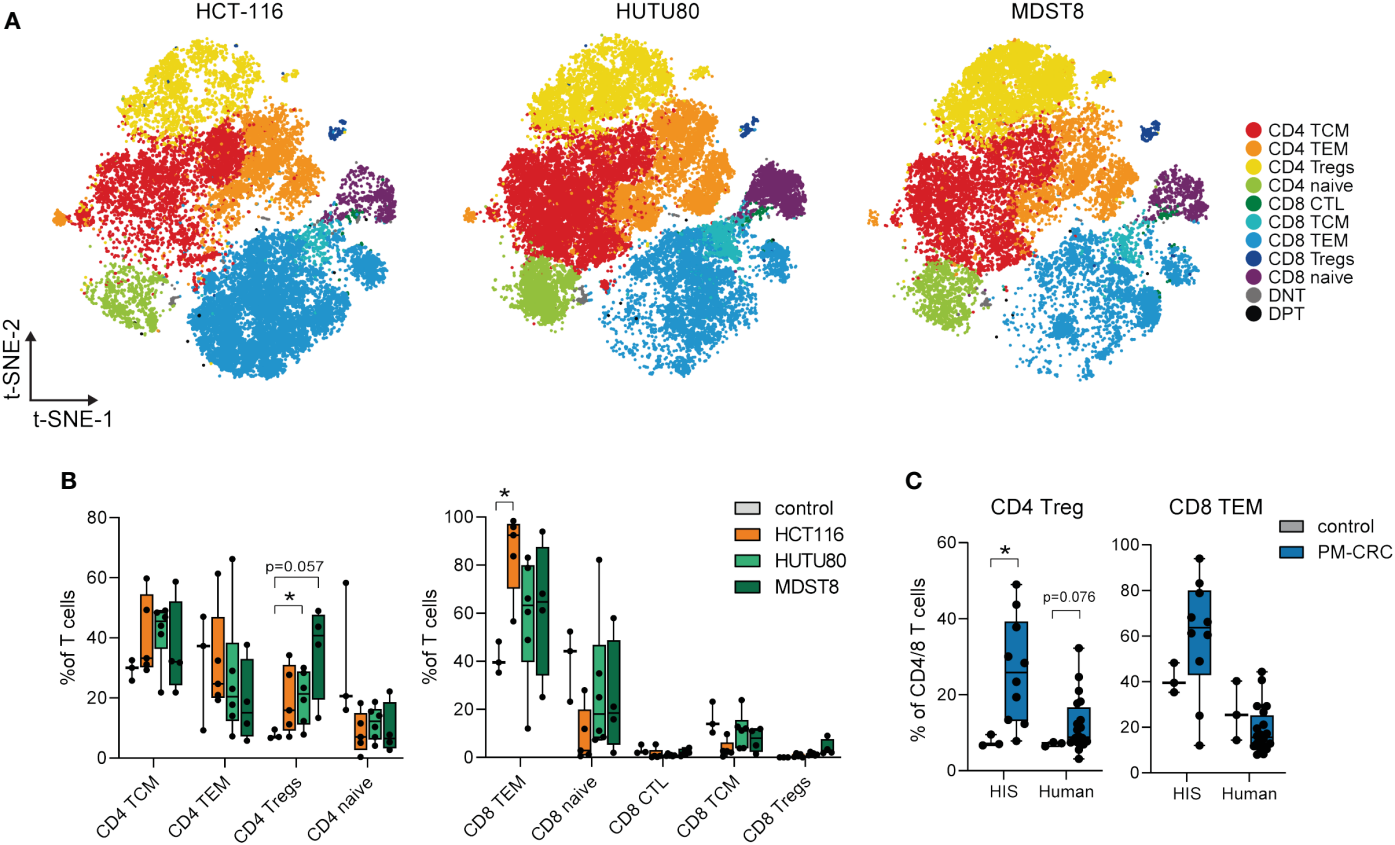
Peritoneal Tregs in the PF of HIS mice expand only in MSS PM-CRC, while MSI PM-CRC-responses are defined by CD8 TEM expansion. **(A)** t-SNE of reclustered T cell immune subsets in HIS PF, color coded per immune subset (n=11) and split by cell line. **(B)** Boxplot comparison of PF from HIS control mice (n=3), and mice injected with HCT116 (n=5), HUTU80 (n=6) and MDST8 (n=4), showing proportion of different CD4- and CD8 T cell subsets in the PerIS relative to total CD4T/CD8T cells, grouped per main T cell lineage. Statistics: Mann-Whitney U test. **(C)** Boxplot comparison of PF from both control and PM-CRC of both HIS and human showing proportion of CD4 Tregs and CD8 TEMs. Both HIS (n=10) and human (n=19) samples are exclusively MSS tumor bearing. Statistics: Mann-Whitney U test. HIS, humanized immune system; PF, peritoneal fluid; MSI, microsatellite instable; MSS, microsatellite stable; CD4 T, CD4^+^ T cells; CD8 T, CD8^+^ T cells; TCM, T central memory; TEM, T effector memory; Tregs, regulatory T cells; CTL, cytotoxic T cells; DNT, double negative T cells; DPT, double positive T cells; t-SNE, t-Distributed Stochastic Neighbor Embedding; PM-CRC, peritoneal metastasized colorectal cancer. Whiskers show minimum and maximum data values. * = p ≤ 0.05.

To further investigate whether the T cell responses observed in the PerIS of HIS mice are cell line/MSS status-dependent, we characterized peritoneal T cell responses in all HIS PM-CRC mice (n=15; 246.050 cells). All annotated T cell subsets could be identified in the PerIS of mice with either MSS cell lines HUTU80 and MDST8, or MSI cell line HCT116 (**Figure 3A**; **Supplementary Figure 3A**). Interestingly, the proportional abundance of CD4^+^ Tregs was increased only in the MSS cell lines HUTU80 and MDST8 as compared to control (mean 20.2 and 35.6% versus 7.7%, respectively). Instead, CD8^+^ T effector memory cells (TEMs) were only significantly increased in MSI cell line HCT116 compared to HIS control (mean 85,4% versus 41,0%, respectively) (**Figure 3B**). Further exploration revealed similar expression of canonical markers, identifying similar T cell subsets in both HIS and human (**Supplementary Figures 3B–D**). Interestingly, the observed Treg responses in MSS PM-CRC (MDST8 and HUTU80) of HIS mice was also observed in the human PerIS in the setting of peritoneal metastasized MSS CRC (mean 7,0% vs 12,11% and 7,7% vs 26,5%, respectively (**Figure 3C**) while no changes were observed in CD8^+^ TEMs in PF of HIS mice nor human PF (**Figure 3C**).

Taken together, our findings demonstrate that the proportional distribution of peritoneal T cell subsets in HIS mice mimics that of the human PerIS, and is tumor cell line and MSS status-dependent. This highlights the potential of HIS mice as a pre-clinical model for studying T cell biology within the context of PM-CRC.

### 3.4 Peritoneal metastases in HIS mice are infiltrated by activated CD4 TCMs, CD4 Tregs and CD8 TEMs

**Figure 4:**
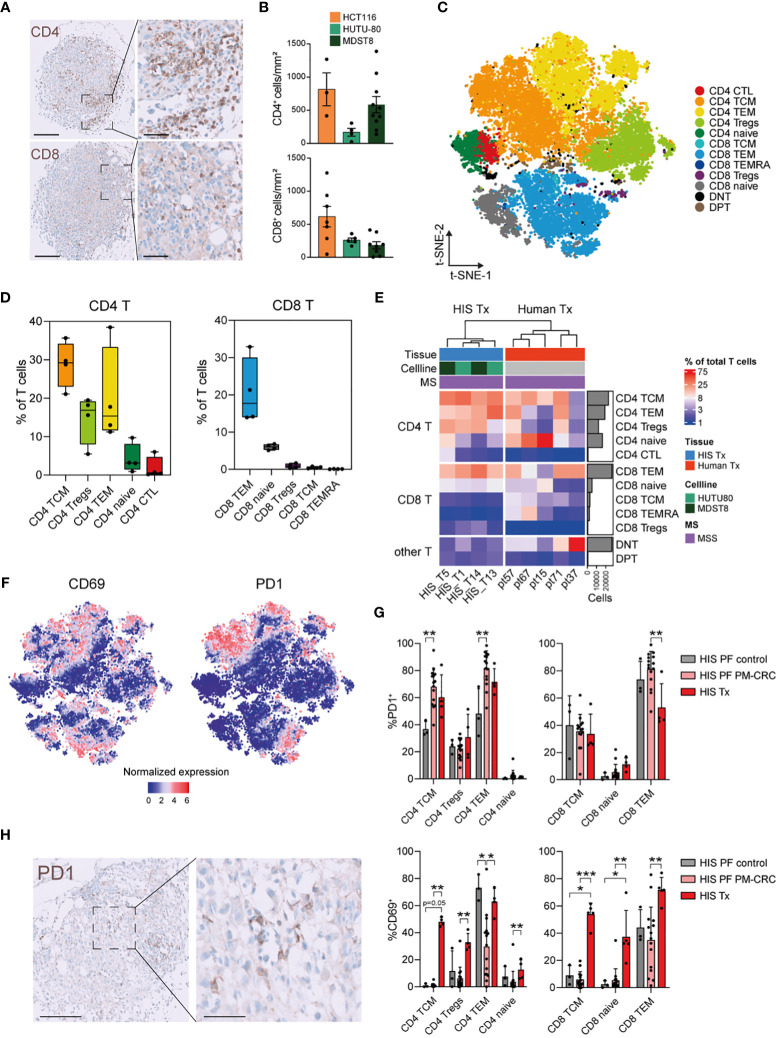
Peritoneal tumors in HIS mice have increased CD4 TCM, CD4 Treg and CD8 TEM subsets which show high expression of CD69 and PD1. **(A)** Immunohistochemical staining of CD4^+^ and CD8^+^ cells infiltrating HIS peritoneal tumors (representative picture: MDST8 cell line) Scale bars 200 µm (left) and 60 µm (right). **(B)** Quantification of infiltrating immune cells per cell line, manually counted using QuPath software. Every dot represents one tumor from the indicated cell line. Bar graph shows mean ± SD of minimally 3 technical replicates. **(C)** t-SNE of reclustered T cell immune subsets in PM of MSS-HIS mice, color coded per immune subset (n=12). **(D)** Boxplot analysis of T cells in PM from MSS-HIS mice (n=4) showing proportion of T cell subsets in the PM relative to T cells, grouped per main T cell lineage (CD4 left; CD8 right). **(E)** Complex supervised heatmap showing the proportional abundance relative to total CD45^+^ immune cells per main T cell subset (CD4 T, CD8 T and other T) of PM of both MSS-HIS mice (n=4) and humans (n=5; MSS only). **(F)** Feature plot showing T cell activation/exhaustion markers on PM derived T cell subsets, CD69 (left) and PD1 (right). **(G)** Bar graph comparison of PD1^+^ (upper panel) and CD69^+^ (lower panel) expressing T cells subsets from PF of control HIS mice, MSS tumor bearing HIS mice and PM (MSS only). Bar graph shows mean ± SD of minimally 3 technical replicates. **(H)** Immunohistochemical staining of PD1^+^ cells that have infiltrated a HIS peritoneal tumor from the MDST8 cell line. Scale bars 0-100um, magnification 5x (left) and 20x (right). HIS, humanized immune system; MSI, microsatellite instable; MSS, microsatellite stable; CD4 T, CD4 T cells; CD8 T, CD8 T cells; TCM, T central memory; TEM, T effector memory; Tregs, regulatory T cells; CTL, cytotoxic T cells; DNT, double negative T cells; DPT, double positive T cells; PD1, Programmed cell death protein 1; t-SNE, t-Distributed Stochastic Neighbor Embedding; PM, peritoneal metastasis. Whiskers show minimum and maximum data values.

To investigate the contribution of T cells to the TIME, we characterized peritoneal tumors using conventional immunohistochemistry and CyTOF. Human CD4^+^ T cells, CD8^+^ T cells, CD20^+^ B cells, and CD68^+^ myeloid cells were identified in the tumors of HIS mice (**Figures 4A, B**; **Supplementary Figures 4A, B**). For CyTOF analysis of HIS tumors, viable DAPI^-^CD45^+^ human immune single cells were isolated from peritoneal tumors (n=4; 75.026 cells) (**Supplementary Figures 4C, D**). Using t-SNE visualization, we identified 11 major human immune cell subsets in the peritoneal tumors (**Supplementary Figure 4E**). Notably, CD4^+^- and CD8^+^ T cells were the most abundant immune cell populations, while myeloid cells were largely absent, as expected. Within their respective T cell, myeloid, B cell an NK cell lineages, CD4^+^ T cells, granulocytes, B naïve and NK CD16^–^ cells comprised the largest subsets, respectively (**Supplementary Figure 4F**). Additionally, we compared canonical marker expression of immune subsets to human peritoneal metastasis samples, confirming similar annotation of human immune cells in HIS mice and humans (**Supplementary Figure 4G**). Reclustering of T cells (n=4; 50.943 cells) found in the peritoneal tumors enabled deeper characterization and led to the identification of 12 different T cell subsets (**Figure 4C**), which could be identified using canonical marker expression (**Supplementary Figure 3B**). Within the CD4^+^ T cells subsets, the most abundant cells were CD4^+^ T central memory cells (TCMs; mean 28,8%), CD4^+^ Tregs (mean 14,7%), CD4^+^ TEMs (mean 20,1%), CD4^+^ naïve (mean 4,2%) and CD4^+^ cytotoxic T cells (CTLs; mean 1,8%) (**Figure 4D**). In the CD8^+^ T cells compartment, CD8^+^ TEMs are the most abundant (mean 20,6%), followed by CD8^+^ naïve (mean 6,0%) cells. CD8^+^ Tregs, CD8^+^ TCMs and CD8^+^ terminally differentiated effector memory cells (TEMRAs) were present only in very low numbers (mean <1,0%) (**Figure 4D**). Interestingly, the distribution of CD8^+^ T cells subsets largely mimicked human tumors (n=5; 91.050 cells), where CD8^+^ TEMs were also the most abundant CD8^+^ T cell subset found (**Figure 4E**). Similar as in PF, peritoneal tumors in HIS mice contained more CD4^+^ Tregs than human tumors (**Supplementary Figure 4H**). As the various immune checkpoints represent key modulators of anti-cancer immunity (17), we investigated the expression of immune activation/exhaustion markers on peritoneal tumor-derived T cells. Both CD69 and PD1 were markedly expressed on different T cell subsets (**Figure 4F**). Interestingly, cells with the highest expression of PD1 were found in either the PF of PM-CRC HIS mice or the tumor (Tx) of HIS mice compared to PF of control HIS mice (**Figure 4G**). Intratumoral PD1 expression was validated using conventional immunohistochemistry (**Figure 4H**). Furthermore, T cell activation/exhaustion, as shown by increased CD69 expression, is more pronounced in Tx of HIS mice compared to PF of either PM-CRC or control HIS mice, allowing for anti-CD69 directed immunotherapies to be studied (**Figure 4G**) (18). In conclusion, the TIME of peritoneal tumors in HIS mice are populated with predominantly CD4^+^ TCMs, CD4^+^ Tregs and CD8^+^ TEMs, and show increased expression of immune-oncology targets compared to their control PerIS counterparts.

## 4 Discussion

This study presents a single cell immune characterization of the PerIS in humans and in a newly developed pre-clinical HIS mouse model for PM-CRC. We achieved successful outgrowth of both MSS and MSI peritoneal CRC lines in the peritoneal cavity of HIS mice, and demonstrated that this evokes MSS status-driven differential immune responses in the peritoneal cavity, which resemble human PerIS responses in PM-CRC. Notably, only PM derived from HUTU80 and HCT116 cell lines were linked to the development to ascites in the mice, potentially correlating with a higher PCI score (**Supplementary Figure 2A**).

One of the important findings from this study is that the composition of peritoneal T cells in NSG mice, after intrahepatic administration of CD34^+^ HSC, resembles the human T cell compartment in the peritoneal cavity. This enables the study of T cell biology in an *in vivo* preclinical model, and to test novel T cell directed targeted therapies. In addition, we intriguingly found that both in human and HIS mice, the peritoneal CD4^+^ Treg subset expands in MSS PM-CRC. Furthermore CD8^+^ TEMs increase in MSI-HIS PM-CRC. Indeed, the TIME in human MSS CRC shows expanded CD4 Treg population (19–22).

Tregs are a subset of CD4^+^ T cells which ensure peripheral self-tolerance having escaped thymic negative selection (21). While critical for immune homeostasis, the presence of Tregs poses a potential hindrance to protective anti-tumor immunity. There is substantial evidence that this function of Tregs is exploited by several cancers to escape immune surveillance. Correspondingly, Tregs are present in the tumor microenvironment of many solid tumors and their accumulation is associated with poor outcomes (21–24). It has been shown that Tregs elicit tissue-specific functions and unlike in several metastatic diseases, the role of Tregs in peritoneal metastasis remains elusive (25).

Next to T cell subsets, we also investigated the activation/exhaustion status of T cell subsets in PM-CRC. Furthermore, out of all T cells within the TIME, CD4^+^ TCMs, CD4^+^ TEMs and CD8^+^ TEMs are the most activated/exhausted subsets, indicated by the increased expression of CD69 and PD1, which is associated with hampered anti-tumor response (26, 27). This dense T cell infiltrate allows for further exploration of emerging adoptive T cell therapies including tumor-infiltrating lymphocytes (TIL) and chimeric antigen receptor (CAR) based treatments (28). Unfortunately, expression levels of both Tim-3 and Lag-3, upregulated on dysfunctional T cells, were below background levels and could thus not be used for further phenotyping (29).

In a recent study, HIS mice with human metastatic MSI CRC cell lines were generated to study the effect of the local immune environment on the effectiveness of ICB. In this model, PMs do not response to conventional (anti-PD-1 or anti-CTLA-4) ICB therapy, in contrast to for example liver metastasis, because PM lack B cells and tertiary lymphoid structures (TLS) (12). Although we acknowledge the relevance of TLS in the response to immunotherapy, we did not characterize TLS, as it was not the focus of the current study (30, 31). In addition, it is questionable whether this HIS model is suitable to study B cell responses to peritoneal metastases in a pre-clinical setting, as there is a major discrepancy between peritoneal B cell abundance in human and HIS mouse PF.

As expected and consistent with literature, the myeloid lineage reconstitution including monocytes, macrophages and dendritic cells (DCs) was limited in our model (32). Subsequently, although T cell composition in the PerIS of HIS mice is largely similar to the human PerIS, and the PerIS responds in a similar manner to MSS or MSI human cancer cell lines, a disadvantage of this model is the translatability of the myeloid compartment in HIS mice. This is important, as recent studies highlight the dominant presence of macrophages within the human PerIS, and because macrophages may interact with T cells and define their phenotype (9, 33). Potential strategies to overcome this limitation are to transgenically express amongst others granulocyte macrophage colony-stimulating factor in NSG mice (NSG-SMG3) resulting in improved functional human macrophage reconstitution (34, 35). Other disadvantages within patient-derived xenograft models like the HIS model employed here are the limited reconstitution and maturation of human immune cells as well as cross-reactivity to murine epitopes, nicely reviewed elsewhere (36).

In conclusion, this study underscores the value of our HIS mice model to investigate immune cell dynamics, particularly of T cells, in PM-CRC, which may be exploited to test T cell targeted anti-tumor therapies. The presence of known anti-inflammatory cells like CD4 Tregs and the exploration of immune exhaustion markers within the TIME provides insights into potential strategies for therapeutic interventions, especially in patients suffering MSS PM-CRC.

## Data availability statement

The raw data supporting the conclusions of this article will be made available by the authors, without undue reservation.

## Ethics statement

The studies involving humans were approved by the medical ethical committee of the Amsterdam UMC, location Academic Medical Center (AMC) in Amsterdam and were enrolled in the study according to Dutch research guidelines of the Federation of Dutch Medical Scientific Societies (FMDSS), as described in “Human Tissue and Medical Research: Code of Conduct for Responsible use”. Patients did not receive any form of compensation. The studies were conducted in accordance with the local legislation and institutional requirements. The participants provided their written informed consent to participate in this study. The animal study was approved by the animal experimentation committee at the Amsterdam UMC (location Academic Medical Center (AMC) in Amsterdam under the nationally registered license numbers AVD118002016493 and AVD11800202013801). The study was conducted in accordance with the local legislation and institutional requirements. Written informed consent was obtained from the individual(s) for the publication of any potentially identifiable images or data included in this article.

## Author contributions

JS: Conceptualization, Data curation, Formal Analysis, Investigation, Methodology, Software, Validation, Visualization, Writing – original draft. SB: Conceptualization, Data curation, Formal Analysis, Investigation, Methodology, Software, Validation, Visualization, Writing – original draft. JV: Data curation, Formal Analysis, Software, Validation, Visualization, Writing – review & editing. JT: Resources, Writing – review & editing. MW: Methodology, Resources, Supervision, Writing – review & editing. ER: Resources, Writing – review & editing. KL: Conceptualization, Investigation, Methodology, Resources, Writing – review & editing. JG: Resources, Software, Writing – review & editing. LV: Conceptualization, Funding acquisition, Methodology, Project administration, Resources, Supervision, Writing – review & editing. JG: Conceptualization, Funding acquisition, Methodology, Project administration, Resources, Supervision, Writing – review & editing.
