# Supplementary material for: T-cell responses in colorectal peritoneal metastases are recapitulated in a humanized immune system mouse model
Source: Front Immunol. 2024 Jul 9;15:1415457. doi: 10.3389/fimmu.2024.1415457 (PMC11263213; doi:10.3389/fimmu.2024.1415457)
Supplement: Supplementary file 1 [file DataSheet_1.pdf]

		**HC PF**	**CRC-PM PF**	**CRC-PM PM**
No. of cases		3	20	4
Gender	Male	2 (66.67%)	9 (45%)	2 (50%)
	Female	1 (33.33%)	11 (55%)	2 (50%)
Age at surgery		62 (38-65)	64.5 (45-78)	60 (52-70)
BMI		23.2 (22.3-32.1)	26.9 (21.5-33.3)	27.8 (25.5-30.5)
PCI score		N/A	13 (2-39)	11 (4-20)
Prior systemic chemotherapy	<3 months ago	N/A	2 (10%)	0 (0%)
	>3 months ago	N/A	7 (35%)	2 (50%)
	No	N/A	9 (55%)	2 (50%)
MMR status	MMRp	N/A	19 (95%)	4 (100%)
	MMRd	N/A	1 (5%)	0 (0%)
Histology	Adeno	N/A	13 (65%)	3 (75%)
	Mucinous	N/A	6 (30%)	1 (25%)
	Goblet cell	N/A	1 (5%)	0 (0%)

Supplementary Table 1: Baseline characteristics of patients included in the study
For categorical variables, numbers and proportions per subgroup were presented. Continuous variables were described by median and range.

Channel name	Marker	Clone
Y89Di	CD45	HI30
Pr141Di	CD49d	9F10
Ce142Di	CD19	HIB19
Nd143Di	CD127	A019D5
Nd144Di	CD11b	ICRF44
Nd145Di	CD68	Y1/82A
Nd146Di	CD8a	RPA-T8
Sm147Di	CD7	CD7-6B7
Nd148Di	CD66a	CD66a-B1.1
Sm149Di	CD25	2A3
Eu151Di	CD123	6H6
Sm152Di	CD141	1A4
Eu153Di	CD38	HIT2
Sm154Di	CD3	UCHT1
Gd155Di	PD1	EH12.2H7
Gd156Di	CD163	GHI/61
Gd158Di	CD27	L128
Tb159Di	CD11c	3.9
Gd160Di	CD14	M5E2
Dy161Di	CTLA4	14D3
Dy162Di	CD69	FN50
Dy163Di	CD206	152
Dy164Di	CD43	CD43-10G7
Ho165Di	CD45RO	UCHL1
Er166Di	CD44	BJ18
Er167Di	CCR7	G043H7
Er168Di	PDL1	29E.2A3
Tm169Di	CD33	WM53
Er170Di	CD45RA	HI100
Yb171Di	CD9	SN4C3-3A2
Yb172Di	CD57	HCD57
Yb173Di	EpCAM	9C4
Yb174Di	HLA.DR	L243
Lu175Di	LAG.3	11C3C65
Lu176Di	CD4	RPA-T4
Bi209Di	CD16	3G8

Supplementary Table 2: Antibody panel used for cytometry by time of flight (CyTOF). Channel name, marker and clone listed in subsequent columns of a total of 36 surface protein markers

Supplementary Figure 1.

(A) Schematic overview of study outline and patient selection of healthy controls and PM-CRC patients for CyTOF analysis. **(B)** Heatmap showing the median protein marker expression on unsupervised clustered human peritoneal derived T cell subsets. CyTOF = cytometry by time of flight, PM-CRC = peritoneal metastasized colorectal cancer, PF = peritoneal fluid, DNT = double negative T cells, DPT = double positive T cells, TCM = T central memory, TEM = T effector memory, Tregs = regulatory T cells, CTL = cytotoxic T cells, TEMRA = Terminally differentiated effector memory.

Supplementary Figure 2.

(A) Overview of peritoneal tumor locations in HIS mice per injected human CRC cell line, as well as PCI score and ascites formation. **(B)** Representative flow cytometry gating strategy of PF. **(C)** Quantification of sorted live (DAPI$^-$) CD45$^+$ cells from PF. **(D)** t-SNE overlay of HIS PF showing all cells, color coded per injected human cell line: HCT-116 (orange), HUTU-80 (light green) and MDST8 (dark green). **(E)** t-SNE overlay of HIS PF showing all cells, color coded per donor mix used for HIS mice generation. **(F)** t-SNE immune subsets in PF, color coded per immune subset (n=4) and split by condition: HIS (left) Human (right). **(G)** Boxplot analysis of 'other myeloid' cells comparing PF of HIS and Human. **(H)** Heatmap showing the protein marker expression on unsupervised clustered peritoneal derived immune cells of both HIS and human immune cells. **(I)** Boxplot comparison of PF from both HIS control (n=3) compared to human control (n=3) of both CD4T and CD8T cells. HC = healthy controls, PM-CRC = peritoneal metastasized colorectal cancer, PF = peritoneal fluid, CyTOF = cytometry by time of flight, t-SNE = t-Distributed Stochastic Neighbor Embedding, PCI = peritoneal carcinomatosis index, HIS = humanized immune system, CD4 T = CD4$^+$ T cells, CD8 T = CD8$^+$ T cells, Other T = double negative T cells and/or double positive T cells, DCs = dendritic cells, NK = natural killer cell.

Supplementary Figure 3.

(A) Complex unsupervised heatmap showing proportion of T cell immune subsets in the PerIS relative to T cells, grouped per main T cell lineage, of HIS tumor mice (n=15), identifying different injected CRC cell lines and microsatellite status of the tumor. **(B)** Heatmap showing the protein marker expression on unsupervised clustered peritoneal fluid derived T cells of both HIS and human immune cells. **(C)** t-SNE overlay of reclustered T cell immune subsets in the setting of peritoneal metastasis, color coded per origin: HIS PF (blue) and Human PF (red). **(D)** t-SNE of reclustered T cell immune subsets in the setting of peritoneal metastasis, color coded per immune subset (n=11) and split by condition: HIS PF (left) and Human PF (right). HIS = humanized immune system, PF = peritoneal fluid, MSI = microsatellite instable, MSS = microsatellite stable, CD4 T = CD4$^+$ T cells, CD8 T = CD8$^+$ T cells, TCM = T central memory, TEM = T effector memory, Tregs = regulatory T cells, CTL = cytotoxic T cells, DNT= double negative T cells, DPT = double positive T cells, t-SNE = t-Distributed Stochastic Neighbor Embedding.

Supplementary Figure 4.

(A) Immunohistochemical staining of CD20$^+$ and CD68$^+$ cells that have infiltrated a HIS PT from the MDST8 cell line. Scale bars 250 µm (left) and 100 µm (right). **(B)** Quantification of infiltrating immune cells per cell line, manually counted using QuPath software. Every dot represents one tumor from the indicated cell line. **(C)** Representative flow cytometry gating strategy of PT. **(D)** Quantification of sorted live (DAPI$^-$) CD45$^+$ cells from PT. **(E)** t-SNE of PT from MSS-HIS mice (n=4), color coded per immune set (n=11). **(F)** Complex unsupervised heatmap showing the proportional abundance relative to total CD45$^+$ immune cells per main immune lineage (T, myeloid, B and NK) of peritoneal tumors from MSS-HIS mice (n=4). **(G)** Heatmap showing the protein marker

expression on unsupervised clustered PT derived immune cells of both HIS and human immune cells. **(H)** Boxplot analysis of CD4Tregs comparing peritoneal tumors of HIS mice and Human (MSS only). HIS = humanized immune system, PT = Peritoneal Tumor; PF = Peritoneal Fluid, MSS = microsatellite stable, CD4 T = $CD4^{+}$ T cells, CD8 T = $CD8^{+}$ T cells. Other T = double negative T cells and/or double positive T cells, DCs = dendritic cells, NK = natural killer cell, t-SNE = t-Distributed Stochastic Neighbor Embedding.
