# Supplementary figures and images for: T-cell responses in colorectal peritoneal metastases are recapitulated in a humanized immune system mouse model
Source: Front Immunol. 2024 Jul 9;15:1415457. doi: 10.3389/fimmu.2024.1415457 (PMC11263213; doi:10.3389/fimmu.2024.1415457)

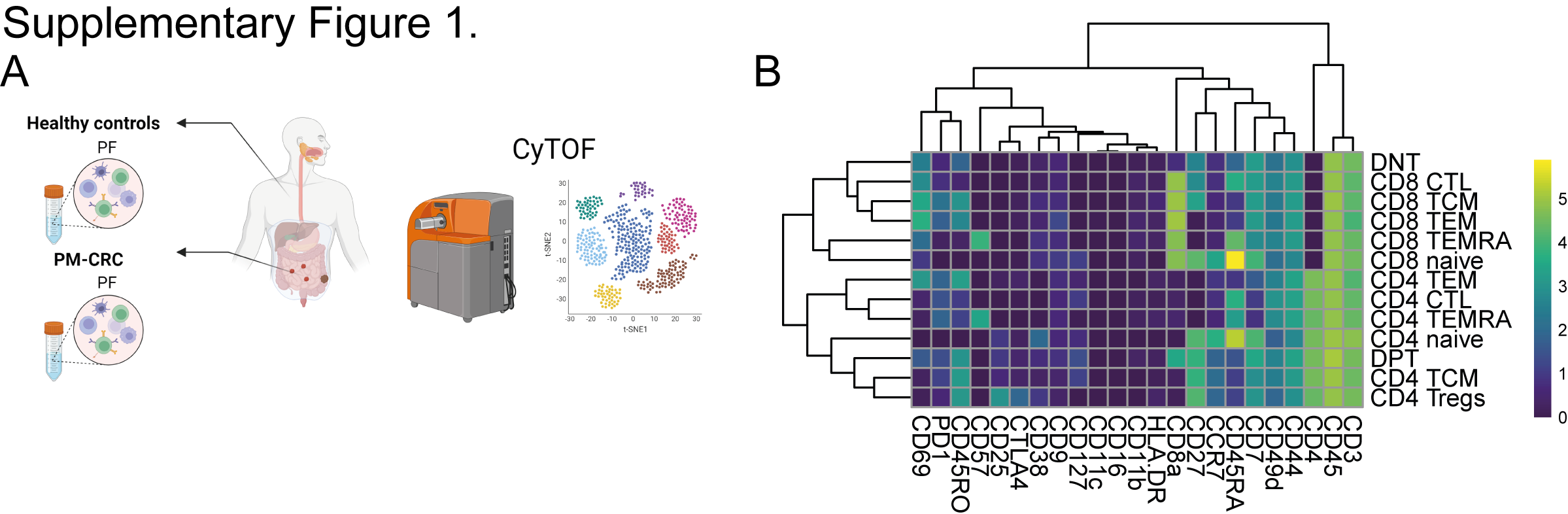

Supplement: Supplementary file 2 [file Image_1.tif]

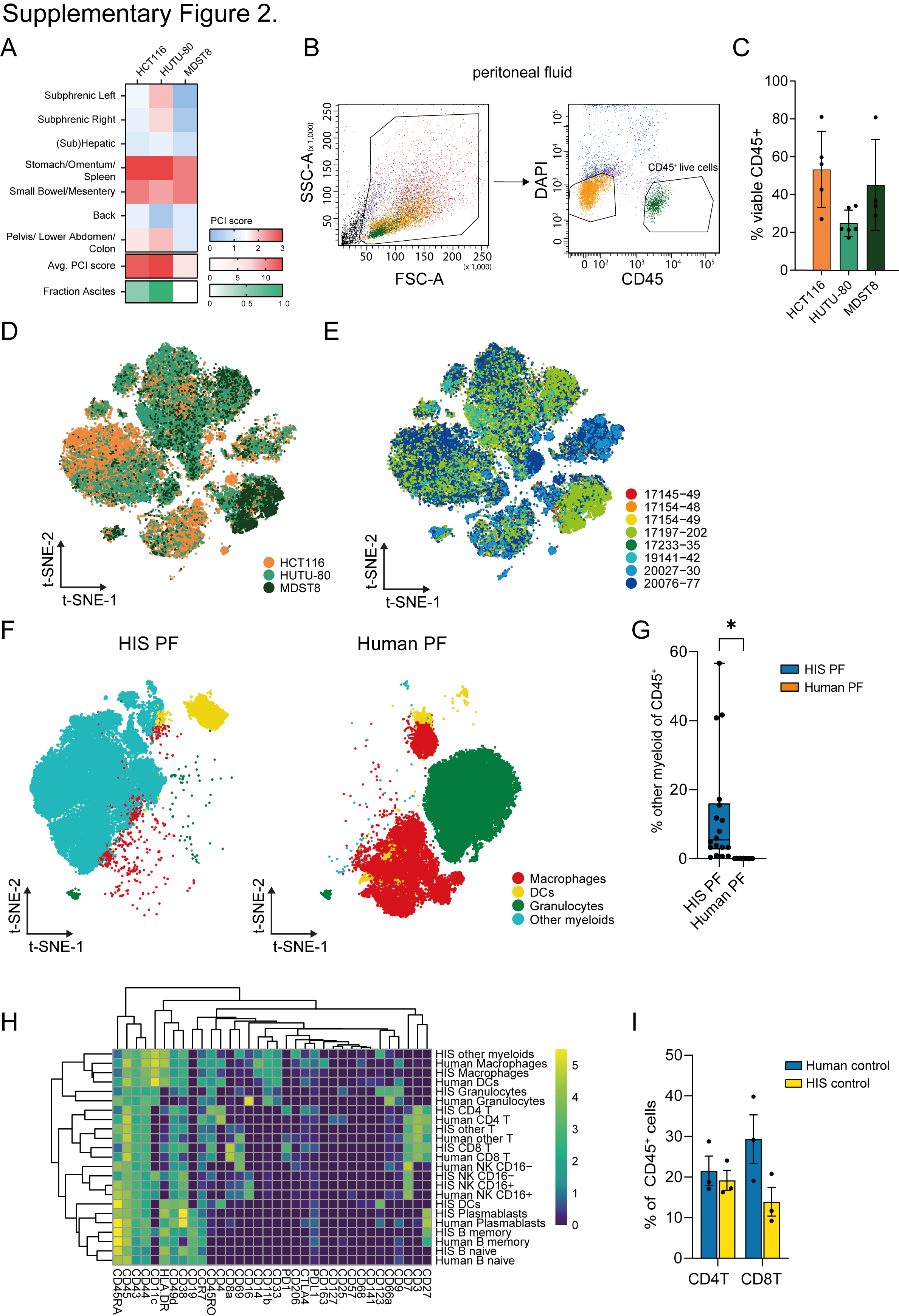

Supplement: Supplementary file 3 [file Image_2.tif]

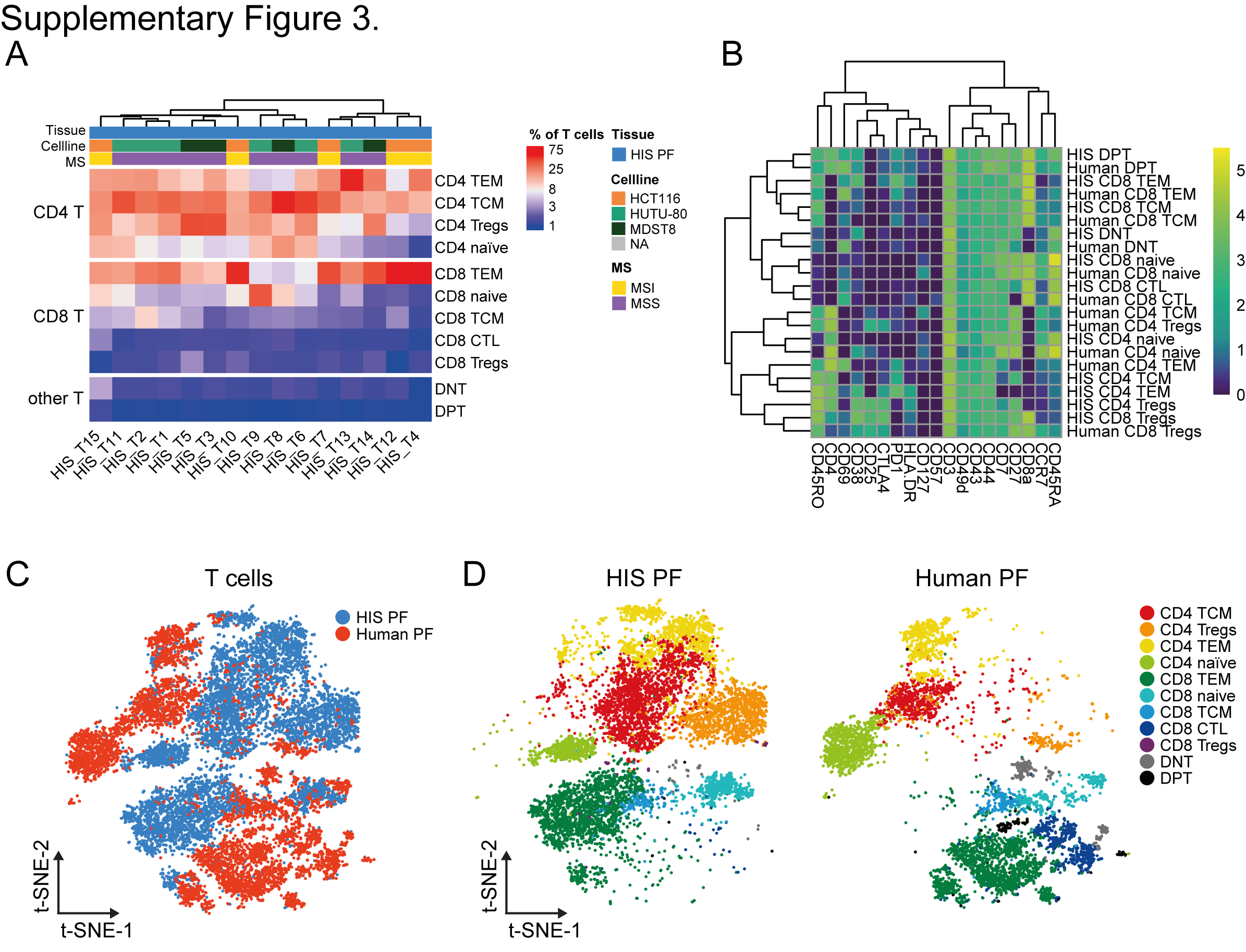

Supplement: Supplementary file 4 [file Image_3.tif]

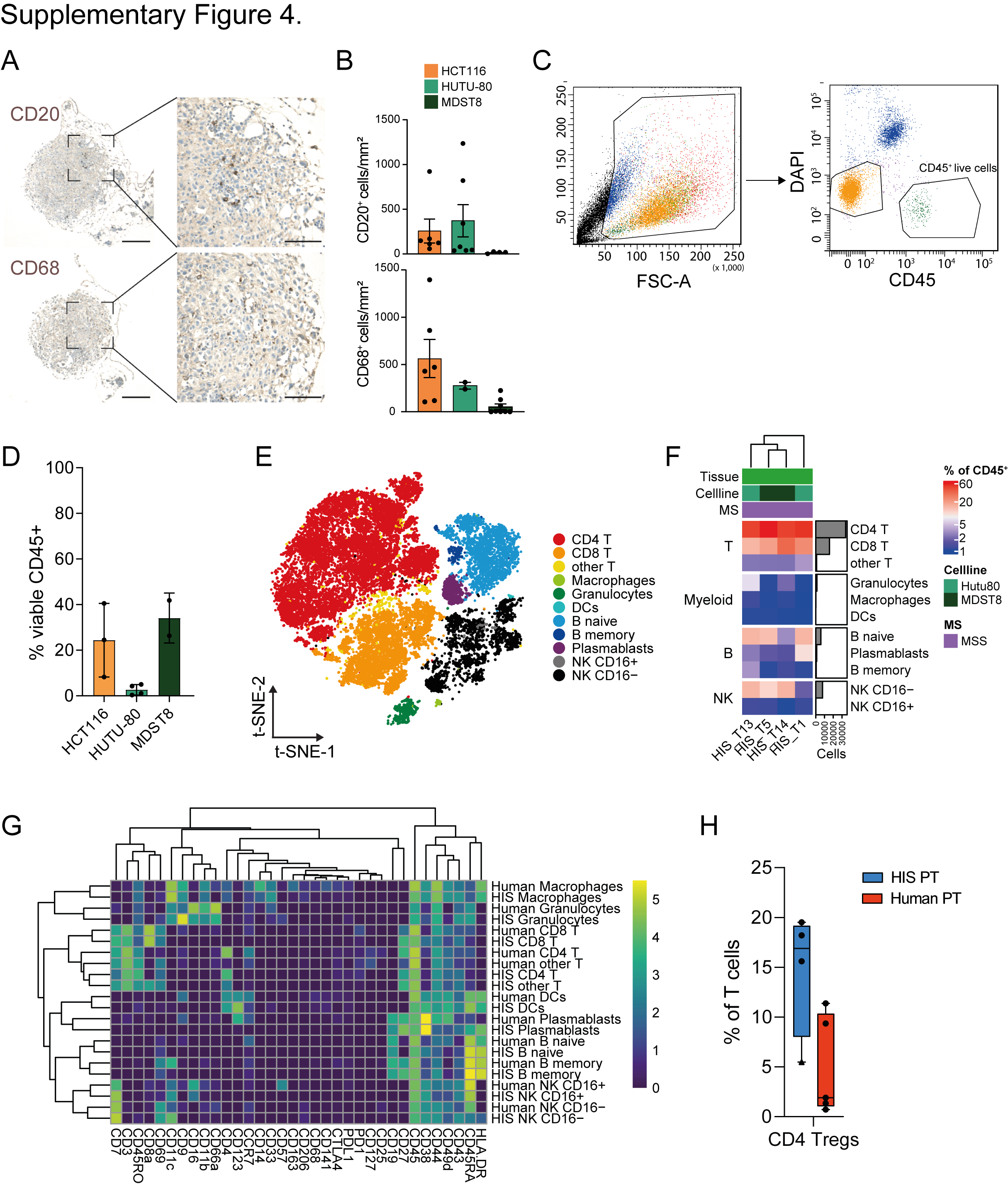

Supplement: Supplementary file 5 [file Image_4.tif]
